# Initial impact of the COVID-19 pandemic on tunisian parents and children

**DOI:** 10.1192/j.eurpsy.2021.722

**Published:** 2021-08-13

**Authors:** R. Manaa, S. Bourgou, N. Kouki, A. Belhadj

**Affiliations:** 1 Child And Adolescent Psychiatry, mongi slim hospital-Tunis, Sousse, Tunisia; 2 Child And Adolescent Psychiatry Department, Mongi slim Hospital-Tunis, Marsa, Tunisia

**Keywords:** adaptation, Behaviour changes, covid 19, security measures

## Abstract

**Introduction:**

Due to the Covid 19 pandemic, the Tunisian government has taken several measures, which had a psychological impact on adults and children.

**Objectives:**

Studying the initial impact of the Covid 19 pandemic on Tunisian parents and children.

**Methods:**

One week after the proclamation of sanitary lockdown in Tunisia, parents were invited to answer voluntarily an anonymous questionnaire posted on social media. The stress level of the parent was measured by the Impact Event Scale Revised (IES-R).

**Results:**

This questionnaire was answered by 138 parents.88% of them were mothers. Changes in working arrangements were made by 87.2% of fathers and 79.8% of mothers. Only 1% of parents did not teach their children about hygienic rules. Parents had a child aged 12 or less in 91% and aged more than 12 in 41.4%. Behavioral changes were reported in 60% for children aged 12 or younger (Graphic 1) and in 20% for those older than 12 (Graphic 2) The IES-R mean score was 28.9 ±18.The severity of the impact was associated with the female sex (p=0.04).We found a positive correlation between the IES-R score and the symptomatology of children with p=0.001 and r=0.518 when the age was superior to 12 and with p<0.001 and r=0,52 when the age was under 12.

**Figure:**
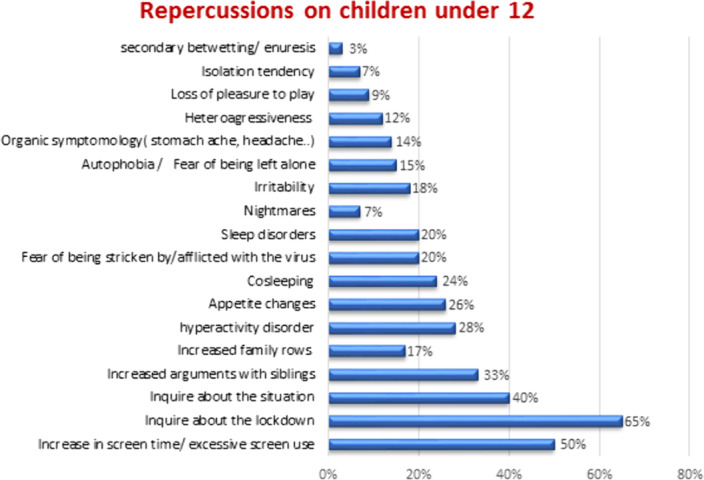

**Figure:**
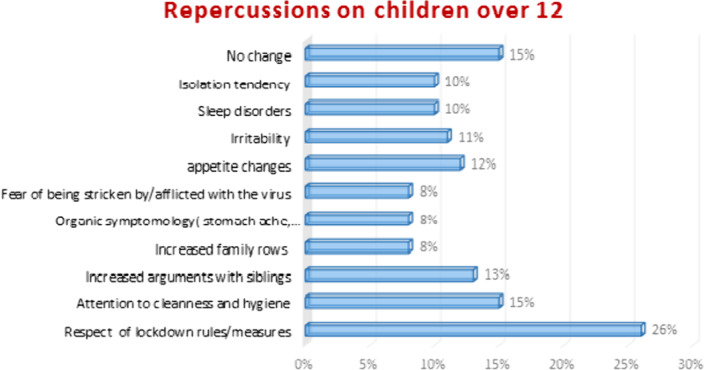

**Conclusions:**

The Covid pandemic in Tunisia affected both parents and children. Psychological intervention is essential to help them get out of this crisis with less damage.

